# Sex-specific metabolic and microbial remodeling in a rotenone-induced rat model of Parkinson’s disease following nicotine administration

**DOI:** 10.1186/s13293-026-00865-1

**Published:** 2026-03-03

**Authors:** Zhen Ni, Gaoge Wang, Qian Li, Xiaqing Wu, Zheng Song, Hao Yu, Pengpeng Yu, Yibo Chen, Lixiang Li, Huan Chen, Hongwei Hou, Qingyuan Hu

**Affiliations:** 1 Beijing Life Science Academy, Beijing, 102209 China; 2 China National Tobacco Quality Supervision &Test Center, Zhengzhou, 450000 China; 3 Key Laboratory of Tobacco Biological Effects, Zhengzhou, 450000 China

**Keywords:** Parkinson's disease, Sex differences, Nicotine, Gut-brain axis, 16S rDNA sequencing, Metabolomics

## Abstract

**Background:**

Parkinson’s disease (PD) is a neurodegenerative disorder with established sex differences in incidence and progression. Epidemiological evidence suggests nicotine may confer protection against PD, but its mechanisms, particularly regarding sex-specific effects, remain unclear. This study investigated the neuroprotective mechanisms of nicotine in a rotenone-induced PD rat model, with a specific focus on evaluating sex-dependent modulation across behavioral, pathological, and gut-related outcomes.

**Methods:**

Male and female Sprague-Dawley rats were administered rotenone (2 mg/kg/day, s.c.) for four weeks to induce PD. Nicotine (0.5 mg/kg/day, s.c.) was administered 30 min after rotenone. Motor function was assessed using rotarod and CatWalk XT gait analysis. Neuropathology in the substantia nigra was evaluated via immunofluorescence for α-synuclein and tyrosine hydroxylase (TH). Gut pathology was analyzed through colon histopathology (H&E staining) and ELISA for IL-6 and α-synuclein. Gut microbiota composition was assessed by 16 S rDNA sequencing, and serum metabolomics was performed using UPLC-MS/MS. Data were analyzed by two-way ANOVA with Tukey’s post-hoc test.

**Results:**

Nicotine significantly attenuated rotenone-induced motor impairments: males showed a superior response in balance-related parameters, while females exhibited enhanced efficacy in dynamic gait metrics. Pathologically, nicotine reduced nigral α-synuclein accumulation and TH depletion in both sexes, with males showing greater α-synuclein accumulation following rotenone exposure. Crucially, nicotine exclusively ameliorated colon histopathology, reduced plasma α-synuclein, and suppressed colon IL-6 in females, while attenuating intestinal α-synuclein accumulation in both sexes. Microbiota analysis revealed sex-divergent taxonomic shifts with nicotine treatment. Metabolomics showed significantly more extensive metabolic reprogramming in females, particularly affecting indole derivatives. Pearson correlations revealed significant sex-specific associations between altered serum indole derivatives and gut microbiota genera.

**Conclusions:**

Nicotine exerts neuroprotection in PD through sex-dependent modulation of multiple pathological pathways, primarily involving the gut-microbiota-metabolite axis. Females benefit from enhanced gastrointestinal protection and metabolic reprogramming, while males show preferential motor balance restoration. These findings underscore the critical importance of sex-stratified therapeutic strategies for PD.

**Supplementary Information:**

The online version contains supplementary material available at 10.1186/s13293-026-00865-1.

## Abstract (plain language summary)

Parkinson’s disease (PD) is a neurodegenerative disorder characterized by motor deficits and the loss of dopamine-producing neurons. Epidemiological studies suggest that nicotine, a primary component of tobacco, may lower the risk of developing PD, with a potentially stronger effect in men. This study aimed to uncover the mechanisms behind this protection, specifically investigating the role of sex differences. Using a rotenone-induced rat model of PD, we evaluated the effects of chronic nicotine administration on motor function, brain pathology, gut health, microbiome composition, and blood metabolites. Our findings reveal that nicotine provides neuroprotection in a highly sex-specific manner. While it improved motor impairments in both sexes, males showed superior recovery in balance-related parameters, whereas females exhibited greater enhancement in dynamic gait metrics. In the brain, nicotine reduced the accumulation of the pathological protein α-synuclein and protected neurons in both sexes. Crucially, nicotine modulated multiple components of the gut–brain axis in a sex-dependent manner: it significantly improved colon health, reduced plasma α-synuclein, and suppressed gut inflammation in females, whereas corresponding changes in males were less pronounced or did not reach statistical significance. Microbiome and metabolomic analyses further indicated sex-biased patterns of response. Females experienced more extensive metabolic reprogramming, particularly in indole derivatives, which was linked to specific gut bacteria. In contrast, males showed restoration of distinct bacterial genera but had a weaker metabolic response. Collectively, these findings suggest that nicotine engages the gut–microbiota–metabolite axis in both sexes, with differences in the relative contribution and magnitude of individual pathways, underscoring the importance of considering sex as a biological variable in PD research and therapeutic development.

**Supplementary Information:**

The online version contains supplementary material available at 10.1186/s13293-026-00865-1.

## Highlights

- Nicotine administration significantly attenuated rotenone-induced motor deficits, with sex-dependent differences in specific motor outcomes.
- Nicotine exerted sex-dependent gastrointestinal effects.
- Nicotine treatment selectively restored beneficial genera in a sex-dependent manner, normalizing *Romboutsia* in females and butyrate-producing genera *Blautia* and *Marvinbryantia* in males.
- Metabolomic profiling identified significantly more extensive nicotine-mediated metabolic normalization in females, particularly affecting indole derivatives.

**Supplementary Information:**

The online version contains supplementary material available at 10.1186/s13293-026-00865-1.

## Introduction

PD is a progressive neurodegenerative disorder marked by motor symptoms such as tremor, rigidity, bradykinesia, and postural instability [1]. Its hallmark pathological features include the degeneration of dopaminergic neurons in the substantia nigra pars compacta (SNpc), abnormal aggregation of α-synuclein into Lewy bodies, neuroinflammation, and oxidative stress [2]. Current pharmacological treatments, including levodopa and dopamine agonists, provide symptomatic relief but do not halt disease progression and often lead to long-term complications [3].

Increasing evidence implicates the gastrointestinal tract and gut-brain axis in PD pathogenesis and progression. Non-motor symptoms such as constipation and intestinal inflammation frequently precede motor onset by years [4, 5], suggesting peripheral pathology may drive central neurodegeneration. Altered gut microbiota composition is consistently observed in PD patients [6, 8], with specific genera correlating with motor severity, endocrine, and immune pathways [9, 11]. In addition to compositional changes, microbiota-derived tryptophan metabolites (including indole derivatives) can activate the aryl hydrocarbon receptor (AhR), thereby shaping mucosal barrier integrity and inflammatory signaling along the gut–brain axis [12, 13]. AhR signaling has also been implicated in PD-relevant neuroimmune processes and α-synuclein–related pathways in experimental models and mechanistic studies [14]. Notably, gut-derived α-synuclein may propagate to the brain via the gut-brain axis. This is supported by evidence that α-synuclein pathology often originates in the enteric nervous system [15], and that germ-free mice transplanted with microbiota from PD patients develop exacerbated motor deficits [16]. However, the role of sex differences in shaping gut-brain interactions in PD remains poorly characterized.

Epidemiological studies have consistently shown that smokers have a lower risk of developing PD [17], with nicotine, the primary bioactive component in tobacco, implicated as a key mediator. Intriguingly, this protective effect exhibits sex-associated differences [18]: epidemiological data suggest that the inverse association with PD is more pronounced in males [18, 19], mirroring the higher incidence of PD observed in men [20]. Nicotine acts as a nicotinic acetylcholine receptor (nAChR) agonist with established neuroprotective, anti-inflammatory, and antioxidant properties [21, 23]. Critically, emerging evidence indicates nicotine can remodel gut microbiota composition in a sex-dependent manner [24], potentially modulating gut-brain signaling. Given the emerging role of sex chromosomes in mediating male vulnerability to gut-initiated PD pathogenesis [15, 25], nicotine’s sex-specific impact on the gut-brain axis warrants further exploration. A systematic evaluation of nicotine’s sex-specific effects on central pathology, peripheral inflammation, gut integrity, and the microbiota-metabolite axis in PD models is lacking.

In this study, we employed a rotenone-induced rat model of PD in both sexes to investigate sex-dependent therapeutic responses to nicotine. We evaluated motor performance, nigrostriatal and gastrointestinal pathology, as well as gut microbiota composition and host metabolism to explore the therapeutic potential of nicotine and its modulation of the gut-brain axis.

## Materials and methods

### Animals

Male and female Sprague-Dawley rats (7–8 weeks old, body weight 250 g ± 20 g) were purchased from Charles River Laboratories (Beijing, China). Three rats were kept in each cage under conditions of 18–22℃, 40–60% relative humidity, and a 12-hour light-dark cycle. During the housing period, the rats had free access to food and water. All animal treatment procedures adhered to the guidelines of the Laboratory Animal Management and Ethics Committee of the China Tobacco Quality Supervision and Test Center (protocol code: CTQTC-SYXK-2023015).

### Drug treatments

Rotenone (Sigma, R8875) was initially dissolved in DMSO and then diluted with corn oil to a concentration of 2 mg/mL. The administration dose was 2 mg/kg/day via subcutaneous injection [26, 27]. The rats were randomly divided into the following six groups: male control group, male PD group (daily subcutaneous injection of 2 mg/kg rotenone), and male PD + Nic group (subcutaneous injection of 0.5 mg/kg nicotine 30 min after daily rotenone injection); female control group, female PD group (daily subcutaneous injection of 2 mg/kg rotenone), and female PD + Nic group (subcutaneous injection of 0.5 mg/kg nicotine 30 min after daily rotenone injection). The treatment was administered continuously for four weeks.

### Behavioral examination

After 4 weeks of injections, behavioral tests were conducted on the rats. Prior to testing, a five-day training period were implemented to minimize the effects of anxiety and fear.

### Rotarod test

The rats were placed on a rotarod apparatus, which rotated at a constant speed of 12 revolutions per minute for 180 seconds [28]. The animals were allowed to move freely on the rotating rod. The latency to fall off the rod was recorded, and each rat was tested three times. Between each trial, rats were allowed to rest for 30 min.

### Gait analysis

Gait assessment of rats was conducted using the CatWalk XT system (Noldus Information Technology, Wageningen, Netherlands). The animals were allowed to freely explore and walk across a glass walkway, with fluorescent light shining into the glass walkway from one side. In a dim environment, light reflects downwards, and a camera installed under the glass records the footprints of rats walking on the glass walkway. Before the experiment, all rats underwent a five-day training period, with each rat trained three times a day until it was able to cross the track within 10 s, rats that failed to meet this criterion were excluded from the study. Gait data were recorded three times for each rat. Gait training was conducted from day 24 to 28 after rotenone administration, and testing was performed after the completion of the treatment. The gait parameters detected by CatWalk software are described in Supplementary Table 1.

### 16 S rDNA gene sequencing analysis

DNA was extracted from samples according to the manufacturer’s instructions using the MagPure Stool DNA KF Kit B (MAGEN, MD5115-02B). All extraction buffers (including Buffer ATL/PVP-10 and Buffer PCI) were supplied as part of this kit. 100–200 mg of each sample was transferred to a centrifuge tube with grinding beads. A total of 1 mL Buffer ATL/PVP-10 was added, and the sample was homogenized using a grinding machine (Shanghai Jingxin Tech, China), followed by incubation at 65℃ for 20 min. The homogenate was then centrifuged at 14,000×*g* for 5 min (Eppendorf, Germany), and the supernatant was transferred to a new tube. 0.6 mL Buffer PCI was added to the supernatant and mixed thoroughly by vortexing for 15 s. The mixture was centrifuged at 18,000×*g* for 10 min. The supernatant was transferred to a deep-well plate with magnetic beads binding solution (600 µL Buffer with magnetic beads, 20 µL Proteinase K, 5 µL RNase A), followed by sequential additions of 700 µL Wash buffer 1,700 µL Wash buffer 2,700 µL Wash buffer 3, and 100 µL Elution Buffer. The plate was placed into the KingFisher purification system (Kingfisher, Thermo Fisher, USA), and the appropriate automated program was run. Upon completion, purified DNA was transferred to 1.5 mL centrifuge tubes for storage.

Library preparation was performed using 2 × Phanta Max Master Mix (VAZYME, China), and the bacterial 16 S rRNA gene V3–V4 region was amplified using primers 338 F (ACTCCTACGGGAGGCAGCAG) and 806R (GGACTACHVGGGTWTCTAAT). PCR enrichment was performed in a 50 µL reaction containing 30 ng of template DNA and fusion PCR primers. PCR cycling conditions were as follows: 95 °C for 3 min; 30 cycles of 95 °C for 15 s, 56 °C for 15 s, 72 °C for 45 s and final extension at 72 °C for 5 min. PCR products were purified by DNA magnetic beads (BGI, LB00V60). The validated libraries were used for sequencing on Illumina MiSeq/Hiseq platform (BGI, Shenzhen, China) following the standard pipelines of Illumina, and generating 2 × 300 bp paired-end reads.

### Bioinformatic analysis

Raw paired-end reads were quality-filtered to remove low-quality sequences and reads containing ambiguous bases, and then merged to obtain clean tags using PANDAseq (v2.9). Operational taxonomic units (OTUs) were clustered at 97% sequence similarity using an established pipeline [29, 30], and representative sequences were taxonomically assigned using the RDP Classifier (v2.2) against the Ribosomal Database Project database [31].

Alpha diversity (within-sample diversity) was calculated in QIIME (v1.9.1) using the Shannon and Simpson indices [32], where Shannon reflects both richness and evenness and Simpson emphasizes dominance/evenness. Beta diversity (between-sample community dissimilarity) was evaluated using weighted UniFrac distances, which incorporate phylogenetic relationships and account for relative abundance. Principal coordinates analysis (PCoA) was used to visualize beta-diversity distance matrices. Group-level differences in community composition were tested by ANOSIM (analysis of similarities), using permutation-based significance testing. Unless otherwise stated, statistical analyses and visualization were performed in R (V3.5.1).

### Metabolomics

Blood samples were taken from the rats at the end of the behavioral assay. Blood collection was terminal. Rats were anesthetized with isoflurane, and approximately 5 mL of whole blood was collected by orbital exsanguination (retro-orbital blood collection) into procoagulant tubes. The blood was allowed to clot, and serum was then centrifuged at 5,000 rpm at 4 ℃ for 10 min and stored at −80℃. Metabolite separation and detection were performed using a Waters 2777 C UPLC (Waters, USA) coupled with a Q Exactive HF high-resolution mass spectrometer (Thermo Fisher Scientific, USA). Spectrometric conditions: The column used was a BEH C18 column (1.7 μm, 2.1 × 100 mm, Waters, USA). The positive separation mode mobile phases were water containing 0.1% formic acid (Liquid A) and methanol containing 0.1% formic acid (Liquid B), and the negative separation mode mobile phases were water containing 10 mM ammonium formate (Liquid A) and 95% methanol containing 10 mM ammonium formate (Liquid B). The elution was performed using the following gradient: 2% B solution for 0–1 min; 2%−98% B solution for 1–9 min; 98% B solution for 9–12 min; 98% B solution-2% B solution for 12–12.1 min; and 2% B solution for 12.1–15 min. The flow rate was 0.35 mL/min, the column temperature was 45 °C, and the injection volume was 5 µL. Principal component analysis (PCA) was performed using the R package mixOmics (Version 6.32.0) to determine the metabolic differences between different groups of rats. *P*-value and projected variable importance (VIP) of each metabolite were obtained based on partial least squares discriminant analysis model. The two-tailed Student’s t-test was used to calculate the relative levels of metabolites between the two groups. Differential metabolites between groups were determined by *p* < 0.05 and VIP > l.

### Immunofluorescence

The rat brains were fixed in 4% paraformaldehyde for 24 h. The tissues were then embedded in paraffin and serially sectioned into 5-µm-thick coronal sections. Brain and colon sections were dewaxed in xylene and rehydrated through a graded alcohol series. Antigen retrieval was performed on these sections using ethylenediaminetetraacetic acid buffer (pH 8.0) in a microwave, followed by three washes in PBS. After blocking with serum for 1 h, the sections were incubated overnight at 4 °C with the following primary antibodies: Rabbit anti α-synuclein antibody (ab212184; Abcam), Rabbit anti-tyrosine hydroxylase (TH) antibody (ab137869; Abcam), and Mouse anti TLR-4 antibody (ab22048; Abcam). Then, the appropriate secondary antibodies were used to detect the corresponding primary antibodies, including: Goat Anti-Rabbit IgG H&L (ab150078; Abcam), Goat Anti-Chicken IgY H&L (ab150169; Abcam), and Goat Anti-Mouse IgG H&L (ab150119; Abcam). Nuclei were stained with DAPI solution, and images were captured using a fluorescence microscope (Nikon, Tokyo, Japan). For cell identification and quantification, DAPI staining was used to define individual nuclei and to localize cell bodies. Marker-positive cells (e.g., α-synuclein- or TH-positive cells) were defined as DAPI-associated cells exhibiting specific immunofluorescence signals above background levels. Images were acquired using identical microscope settings across experimental groups. The number of positive cells was analyzed using Image-Pro Plus 6.0 software, with three images selected from each slide for calculation.

### Colon histopathology

The colon histopathology method was adapted from Tian Y et al. [33], after the animals were euthanized, the colon was fixed in 10% neutral buffered formalin (Solarbio, China), dehydrated by a graded ethanol series, and paraffin-embedded, Sections were then stained with hematoxylin and eosin (H&E) dyes in accordance with the standard procedure, and observed by light microscope. Pathologic scoring: (1) Ulceration: presence of ulceration = 1 point; absence = 0 points. (2) Intestinal crypt damage (crypt loss): crypt damage was scored based on the estimated proportion of crypts that were lost or destroyed (“crypt dropout”) within the affected mucosal area (relative to adjacent intact mucosa), as follows: 1 point (mild) = approximately 1/3 crypt loss; 2 points (moderate) = approximately 2/3 crypt loss; 3 points (severe) = near-complete crypt loss/absence (approximately 3/3). Here, “1/3, 2/3, and 3/3” refer to the fraction of crypt structures missing/disrupted, rather than a “reduction of crypt damage.” (3) Other lesion severity was scored semi-quantitatively as follows: 0 points (Normal/No lesions); 0.5 points (Minimal/Very slight amount); 1 point (Mild/Slight amount); 2 points (Moderate); 3 points (Marked/Severe/Multiple lesions); 4 points (Extensive/Very severe/Large amounts). The scores for all lesions in each animal were summed to produce a total histopathology score per animal within each group. Higher total scores reflected more severe damage.

### Enzyme-linked immunosorbent assay

The levels of interleukin IL-6, and α-synuclein in the colon and serum of rats were measured using commercially available enzyme-linked immunosorbent assay (ELISA) kits (mlbio Biotechnology, Shanghai, China). Subsequently, the concentrations of these cytokines were determined according to standard curves generated with purified protein standards.

### Statistics and reproducibility

GraphPad Prism 8 was used for statistical analysis and the data were presented as mean ± SEM. A two-way analysis of variance (ANOVA) with Tukey’s post-hoc test was used to determine significant differences in the effects of the group and sex variables on the dependent variable. Correlation analyses were performed using Pearson’s correlation coefficient. Statistical significance was expressed as a p value, and *p* < 0.05 was considered statistically significant. Sample sizes (n) for each experiment are provided in the corresponding figure legends.

## Results

### Sex-specific motor rescue by nicotine in rotenone-induced PD rats

Motor impairment is a core feature of PD and can be reliably reproduced in rodents using the neurotoxin rotenone. To assess the neuroprotective and sex-specific effects of nicotine, male and female rats were administered rotenone daily for four weeks. Nicotine (0.5 mg/kg/day, i.p.) was injected 30 min after each rotenone dose.

**Fig. 1:**
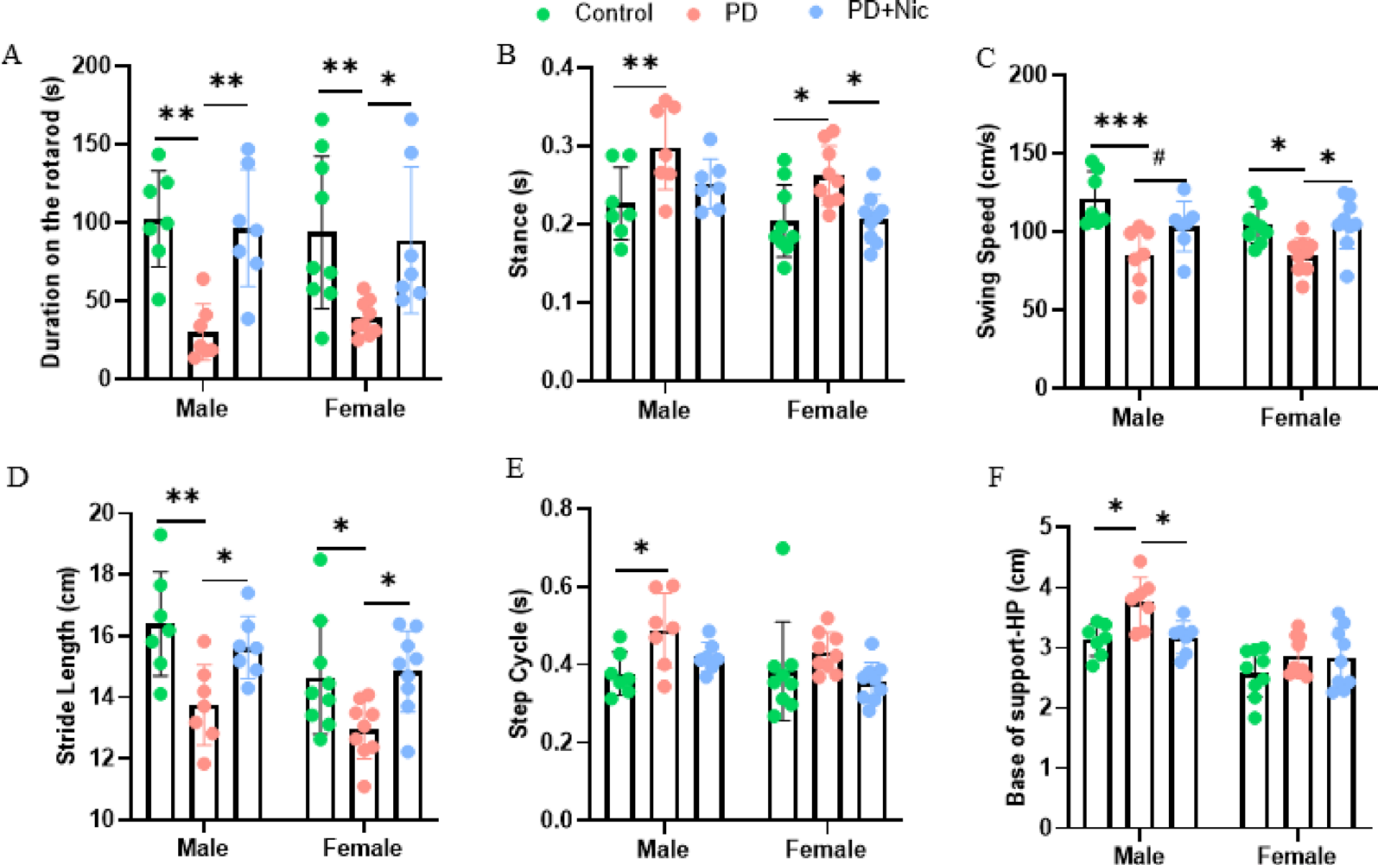
Sex-specific motor deficits and nicotine’s neuroprotective efficacy in rotenone-induced Parkinson’s disease rats. **A** Rotarod performance. **B-F** Parameters of the CatWalk analysis for different groups of rats after four weeks of treatment. **B** Stance duration. **C** Swing speed. **D** Stride length. **E** Step cycle. **F** Hindpaw base of support. *n* = 7–9 for each group. All the data were displayed as mean ± SEM, and the statistical analysis was performed using two-way Anova, #*p* < 0.1, *p < 0.05, **p < 0.01, ***p < 0.0001. HP, hindpaws

Rotarod performance showed a strong group effect (F(2,40) = 15.64, *p* < 0.0001), and nicotine significantly improved performance relative to PD in both males (*p* = 0.0029) and females (*p* = 0.0203) (Fig. 1A). CatWalk gait analysis indicated that rotenone broadly disrupted gait in both sexes, while nicotine produced parameter-dependent recovery, with generally similar directional effects across males and females (Fig. 1B–F). PD increased stance duration in both males (*p* = 0.0067) and females (*p* = 0.0143), and nicotine significantly reduced stance time versus PD in females (*p* = 0.0202) (Fig. 1B). PD also reduced swing speed in males (*p* = 0.0002) and females (*p* = 0.0217), with nicotine significantly improving swing speed in females (*p* = 0.0142) (Fig. 1C). Stride length was significantly reduced by PD in both sexes (males *p* = 0.0028; females *p* = 0.0347) and was significantly restored by nicotine in both males (*p* = 0.0430) and females (*p* = 0.0161) (Fig. 1D). Step cycle was significantly increased by PD only in males (*p* = 0.0269), and nicotine did not significantly normalize this metric in either sex (Fig. 1E). Base of support showed significant main effects of sex (F(1,42) = 28.58, *p* < 0.0001) and group (F(2,42) = 5.79, *p* = 0.0060); PD increased base of support in males (*p* = 0.0108) and nicotine significantly reduced it versus PD in males (*p* = 0.0184), restoring values to a level not different from controls (*p* = 0.9768) (Fig. 1F).

In summary, nicotine significantly attenuated rotenone-induced motor deficits in both sexes. Although males and females showed quantitative differences in baseline impairment and in the extent of improvement across specific motor parameters, the overall therapeutic direction of nicotine was comparable between sexes, indicating sex-dependent modulation of treatment effects.

### Nicotine exerts neuroprotective effects with sex-dependent characteristics against PD pathology in substantia nigra

**Fig. 2:**
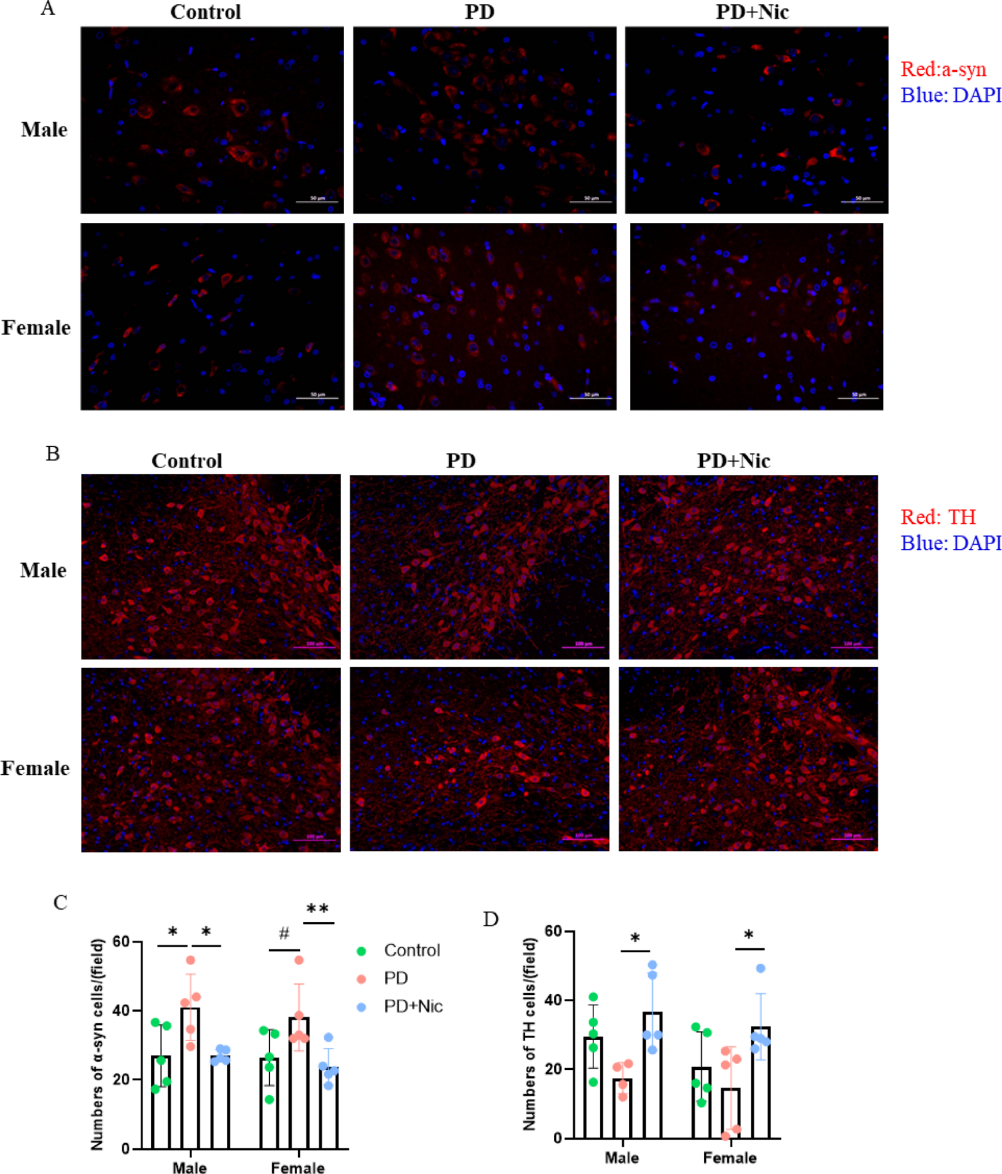
Sex-stratified neuropathological alterations in the SNpc and neuroprotective efficacy of nicotine. **A-B** Representative immunofluorescence images of: **A** α-synuclein (red) with DAPI nuclear counterstain (blue), **B** tyrosine hydroxylase (TH, red), and DAPI (blue) in coronal SNpc sections. **C-D** Quantitative analysis of: **C** α-synuclein-positive inclusions, **D** TH-positive neurons. **A** scale bars = 50 μm; **B** scale bars = 100 μm. Data were presented as mean ± SEM (*n* = 5/group). Statistics (two-way ANOVA with Tukey’s post-hoc test): #*p* < 0.1, *p < 0.05, **p < 0.01

Pathological hallmarks of PD include dopaminergic neuronal loss in the SNpc and abnormal aggregation of α-synuclein into Lewy bodies. Nicotine administration exerted significant neuroprotective effects against rotenone-induced Parkinsonian pathology in both sexes, with sex-dependent differences in the magnitude of changes observed across specific SNpc biomarkers, as assessed by immunofluorescence analysis (Fig. 2A-B). Statistical analysis of α-synuclein immunofluorescence in the striatum revealed a highly significant main effect of group (F_(2,24)_ = 10.17, *p* = 0.0006), whereas neither sex (F_(1,24)_ = 0.621, *p* = 0.438) nor the group × sex interaction (F_(2,24)_ = 0.094, *p* = 0.911) showed significant effects. Post-hoc Tukey tests indicated a numerically larger increase in α-synuclein levels in male PD rats, whereas female PD rats showed a similar but non-significant upward trend (*p* = 0.066). Nicotine treatment significantly reduced α-synuclein accumulation in both sexes compared with untreated PD rats, effectively restoring levels to control values in males and females (Fig. 2C). Statistical analysis of tyrosine hydroxylase (TH) immunofluorescence in the striatum revealed a significant main effect of group (F_(2,23)_ = 8.252, *p* = 0.0020), whereas neither sex (F_(1,23)_ = 2.085, *p* = 0.1622) nor the group × sex interaction (F_(2,23)_ = 0.2294, *p* = 0.7968) reached statistical significance. Post-hoc Tukey’s comparisons indicated that both male and female PD rats exhibited reduced TH levels compared to controls, although these reductions did not reach statistical significance. Crucially, nicotine treatment significantly attenuated TH depletion in PD rats of both sexes compared to untreated PD counterparts, with TH levels restored to values comparable to controls (Fig. 2D). In summary, although male PD rats displayed a numerically greater increase in α-synuclein accumulation following rotenone exposure, this difference did not constitute a statistically supported sex effect. Importantly, nicotine exerted comparable dopaminergic neuroprotection in both sexes.

### Nicotine improves gut pathology and modulates inflammatory markers in PD rats

**Fig. 3:**
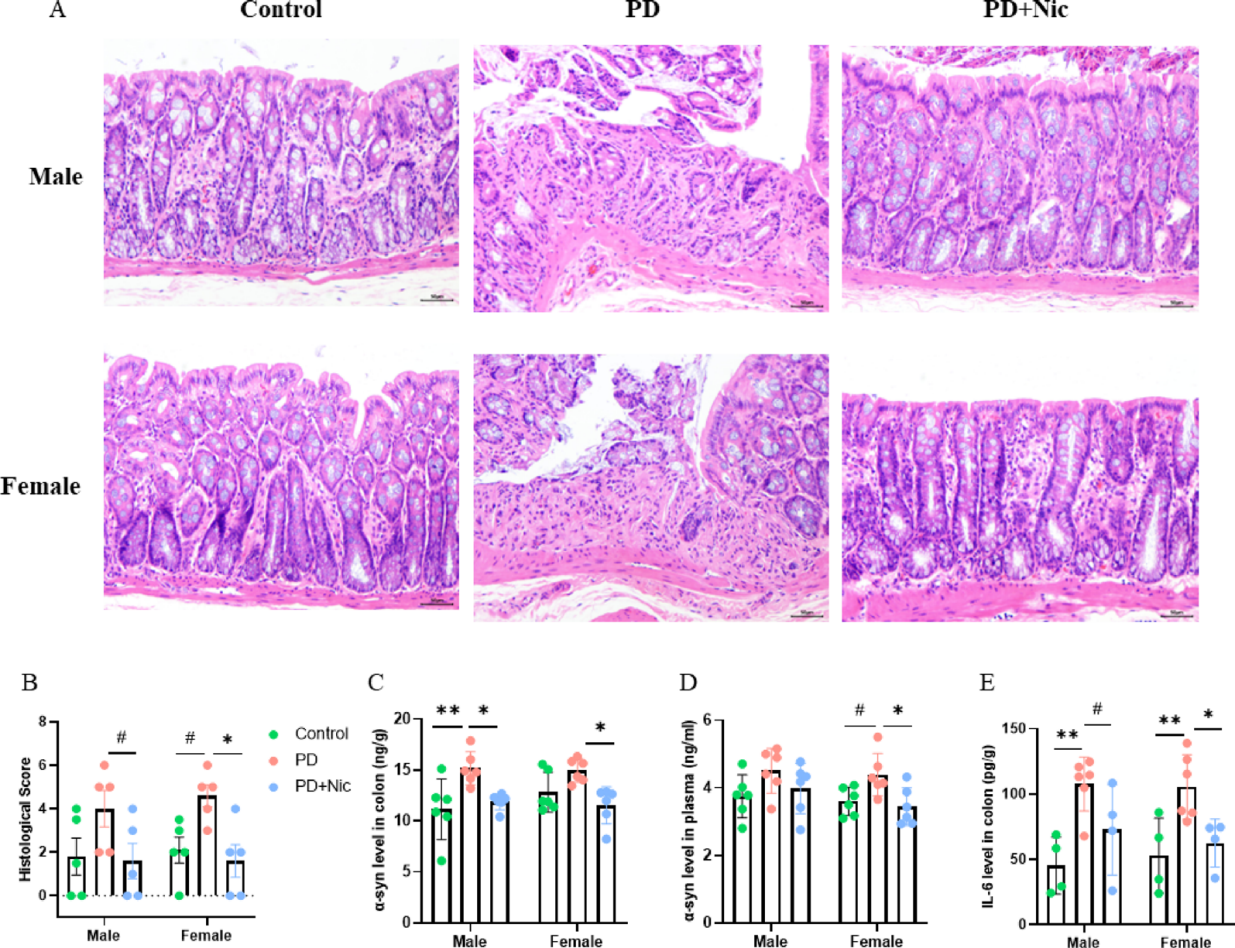
Sex-stratified gut pathology in PD rats. **A** Representative H&E-stained colon sections. **B-E** Quantitative analyses: **B** Histopathology scores, **C** Intestinal α-synuclein, **D** Plasma α-synuclein, **E** Colon IL-6 levels. Data were presented as mean ± SEM (*n* = 4–6/group). Statistics: two-way ANOVA with Tukey’s post-hoc test, #*p* < 0.1, *p < 0.05, **p < 0.01. Scale bar = 50 μm

To investigate the potential link between gut pathology and PD, haematoxylin–eosin (HE) staining was performed on colon tissue sections to assess histological damage (Fig. 3A). Histological analysis revealed significant group effects on gut histopathology scores (F_(2,24)_ = 7.948, *p* = 0.0022), with rotenone-induced damage partially rescued by nicotine. Post-hoc analyses indicated a significant improvement in females, whereas males showed a similar but non-significant trend (Fig. 3B). Parallel analyses showed a robust group effect on intestinal α-synuclein levels (F_(2,30)_ = 12.11, *p* < 0.0001) with sex-biased differences in post-hoc responses. Male PD rats exhibited a significant increase in intestinal α-synuclein compared to controls (*p* = 0.0021), which was attenuated by nicotine (*p* = 0.0122). Female PD rats showed a similar but non-significant baseline elevation (*p* = 0.1302), yet displayed a significant reduction following nicotine administration (Fig. 3C). Plasma α-synuclein analysis confirmed significant group effects (F_(2,30)_ = 5.964, *p* = 0.0066) with nicotine significantly reducing levels exclusively in females (*p* = 0.0332 vs. PD), while males showed non-significant change (Fig. 3D). Inflammation assessment revealed elevated colon IL-6 levels in both sexes after rotenone treatment, with significant group effects (F_(2,22)_ = 13.90, *p* = 0.0001). However, nicotine significantly suppressed IL-6 solely in females (*p* = 0.0364), with males showing a modest, non-significant decrease (Fig. 3E). In summary, nicotine modulated gastrointestinal pathology in a sex-dependent manner. It significantly improved colon histopathology, reduced plasma α-synuclein, and suppressed colon IL-6 in females, while attenuating intestinal α-synuclein accumulation in both sexes, with males exhibiting more severe baseline pathology.

### Sex-specific remodeling of gut microbiota by nicotine in PD rats

**Fig. 4:**
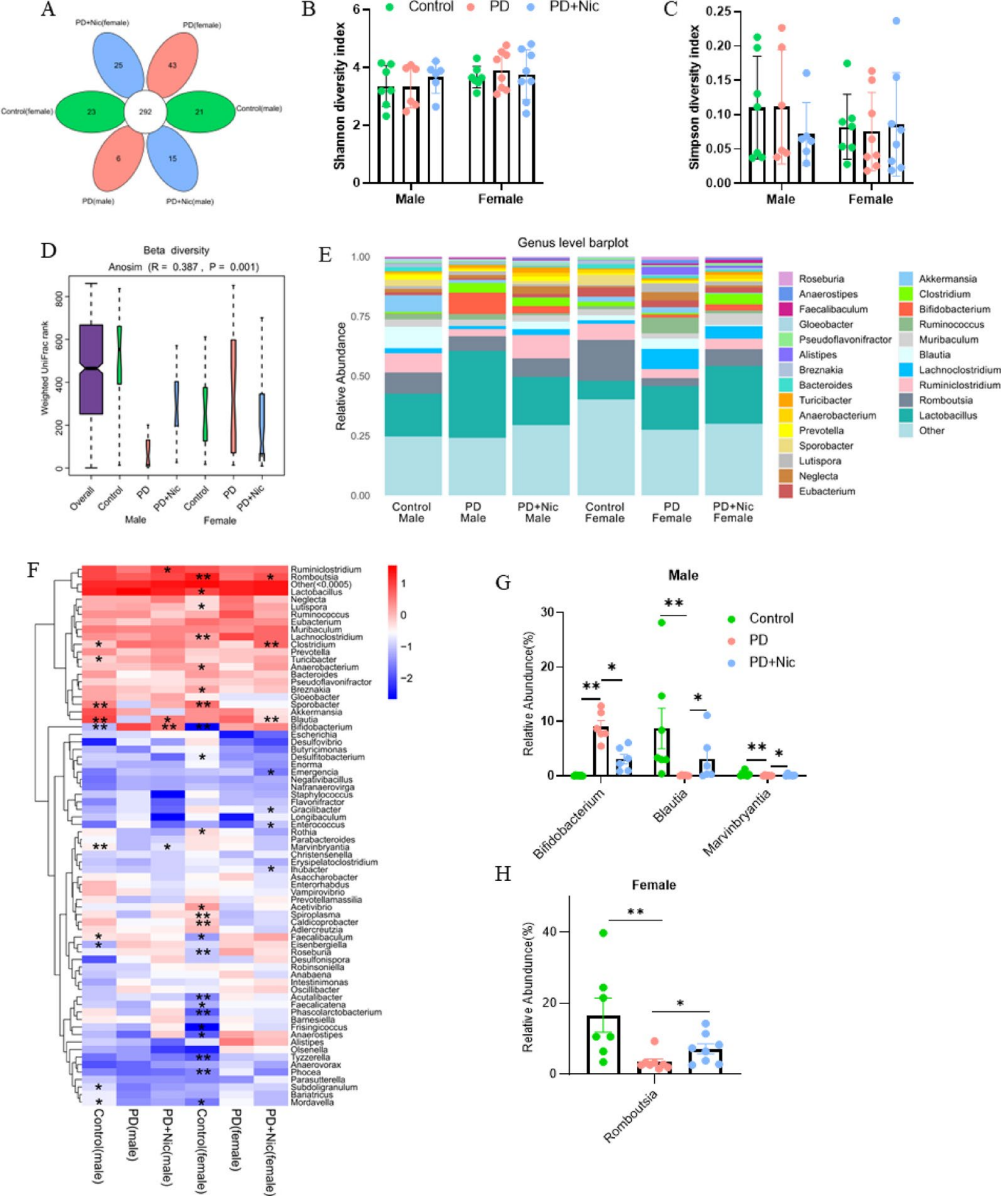
Sex-specific remodeling of gut microbiota by nicotine in PD. **A** Summary diagram showing the number of unique OTUs in each experimental group. **B**,** C** α-Diversity of gut microbiota. **D** β-Diversity analysis. **E** Relative abundance of gut bacteria at the genus level among the six groups. **F** Heatmap showing relative abundance of gut bacteria at the genus level; asterisks denote significant differences compared with the PD group within the same sex. **(G**,** H)** Nicotine ameliorates altered bacterial abundance in male **(G)** and female **(H)** PD rats. Data were presented as mean ± SEM. Statistics: Wilcox.test, *p < 0.05, **p < 0.01, *n* = 6–8 for each group

These sex-dependent alterations in gut integrity, α-synuclein dynamics, and inflammatory responses prompted further investigation into gut microbiome composition, as microbial communities regulate intestinal barrier function and inflammatory pathways in a sex-dependent manner. Characterizing taxonomic shifts associated with the observed sex differences may help elucidate the role of the microbiome in nicotine-mediated gut protection. We analysed the microbial composition of rat faecal samples using 16 S rDNA sequencing (Supplementary Table 2). A total of 292 operational taxonomic units (OTUs) were identified across the six groups. Among the male groups, the unique OTUs in the control group, PD group, and PD + Nic group were 21, 6, and 15 respectively; in females, the corresponding numbers were 23, 43 and 25 respectively (Fig. 4A). The α-diversity analysis, which was conducted to evaluate the richness and diversity of the bacterial species, indicated that there was no significant difference in the α-diversity of gut microbiota among different groups of rats (Fig. 4B Shannon, and Fig. 4C Simpson). Moreover, β-diversity based on weighted UniFrac distances revealed significant differences in microbial community composition among the six groups (*p* = 0.001). ANOSIM analysis demonstrated clear separation of the male PD group from all other groups, while the male control, male PD + Nic, and all female groups clustered more closely (Fig. 4D).

The differences in microbial communities between PD and control groups suggest the presence of gut microbial dysbiosis in PD rats. To explore the abundance and distribution of gut microbiota among the groups and to investigate the potential bacterial groups contributing to microbial dysbiosis, we examined genus-level relative abundances. Only bacterial genera with an average relative abundance of > 1% across all samples are displayed (Fig. 4E). The heatmap further summarizes these genus-level shifts and highlights that PD is associated with broad dysbiosis across multiple taxa in both sexes, while nicotine treatment is accompanied by partial normalization of several PD-altered genera (Fig. 4F). Furthermore, conserved changes across sexes included a significant increase in *Bifidobacterium* abundance and a significant decrease in *Sporobacter* abundance in PD rats of both sexes (Fig. 4F). Importantly, the genera showing nicotine-associated restoration are not identical between males and females, indicating a sex-dependent pattern of microbiota modulation. In males, at the genus level, *Bifidobacterium* was elevated in PD rats and restored by nicotine, whereas levels of *Blautia* and *Marvinbryantia* were significantly decreased in the PD group and restored following nicotine treatment (Fig. 4G). In females, at the genus level, *Romboutsia* abundance was markedly decreased by PD and significantly restored by nicotine intervention (Fig. 4H).

### Metabolomic profiling reveals sex-specific nicotine modulation in PD rats

**Fig. 5:**
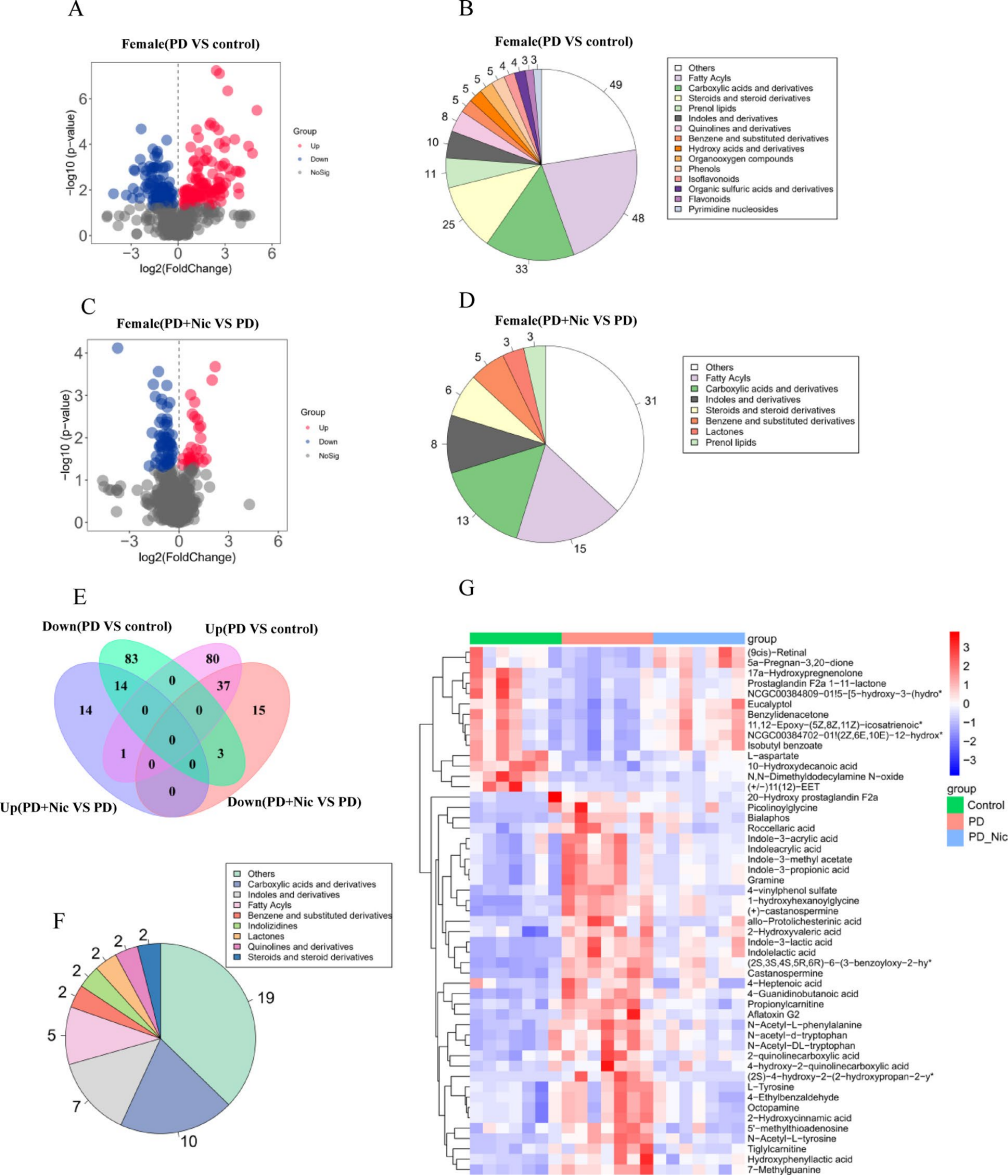
Female-specific metabolic reprogramming by nicotine in PD rats.​​ **A** Volcano plot of differential metabolites between the PD and control groups. **B** Top perturbed metabolite classes between the PD and control groups. **C** Volcano plot of differential metabolites between the PD+Nic and PD groups. **D** Top perturbed metabolite classes between the PD+Nic and PD groups. **E** Venn diagram of metabolite overlap among groups. **F** Class distribution of reversed metabolites. **G** Heatmap of z-score normalized metabolites demonstrating nicotine-mediated normalization of PD signatures. Metabolite names marked with an asterisk are abbreviated due to length, full names are provided in Supplementary Table S3

Ultra-high-performance liquid chromatography-tandem mass spectrometry (UPLC-MS/MS) analysis of serum metabolomes demonstrated more extensive nicotine-associated metabolic changes in females compared to males in rotenone-induced PD rats (Supplementary Table 3). In females, PD induction significantly altered 218 metabolites compared with controls (Fig. 5A), with predominant perturbations in fatty acyls, carboxylic acids/derivatives, steroids/derivatives, and prenol lipids (Fig. 5B). Nicotine treatment further modulated 84 metabolites (29 upregulated, 55 downregulated vs. PD; Fig. 5C), primarily affecting fatty acyls, carboxylic acids/derivatives, and indoles/derivatives (Fig. 5D). Crucially, nicotine reversed 51 PD-induced metabolic alterations (37 suppressed upregulated metabolites, 14 elevated downregulated metabolites; Fig. 5E), predominantly carboxylic acids/derivatives, indoles/derivatives, and fatty acyls (Fig. 5F). Heatmap analysis confirmed nicotine’s capacity to normalize PD-associated metabolic signatures (Fig. 5G). Specifically, nicotine significantly lowered the levels of seven indoles and their derivatives in the PD group: indole-3-lactic acid (ILA), indole-3-methyl acetate, indolelactic acid, indole-3-propionic acid (IPA), indole-3-acrylic acid, indoleacrylic acid, and gramine.

**Fig. 6:**
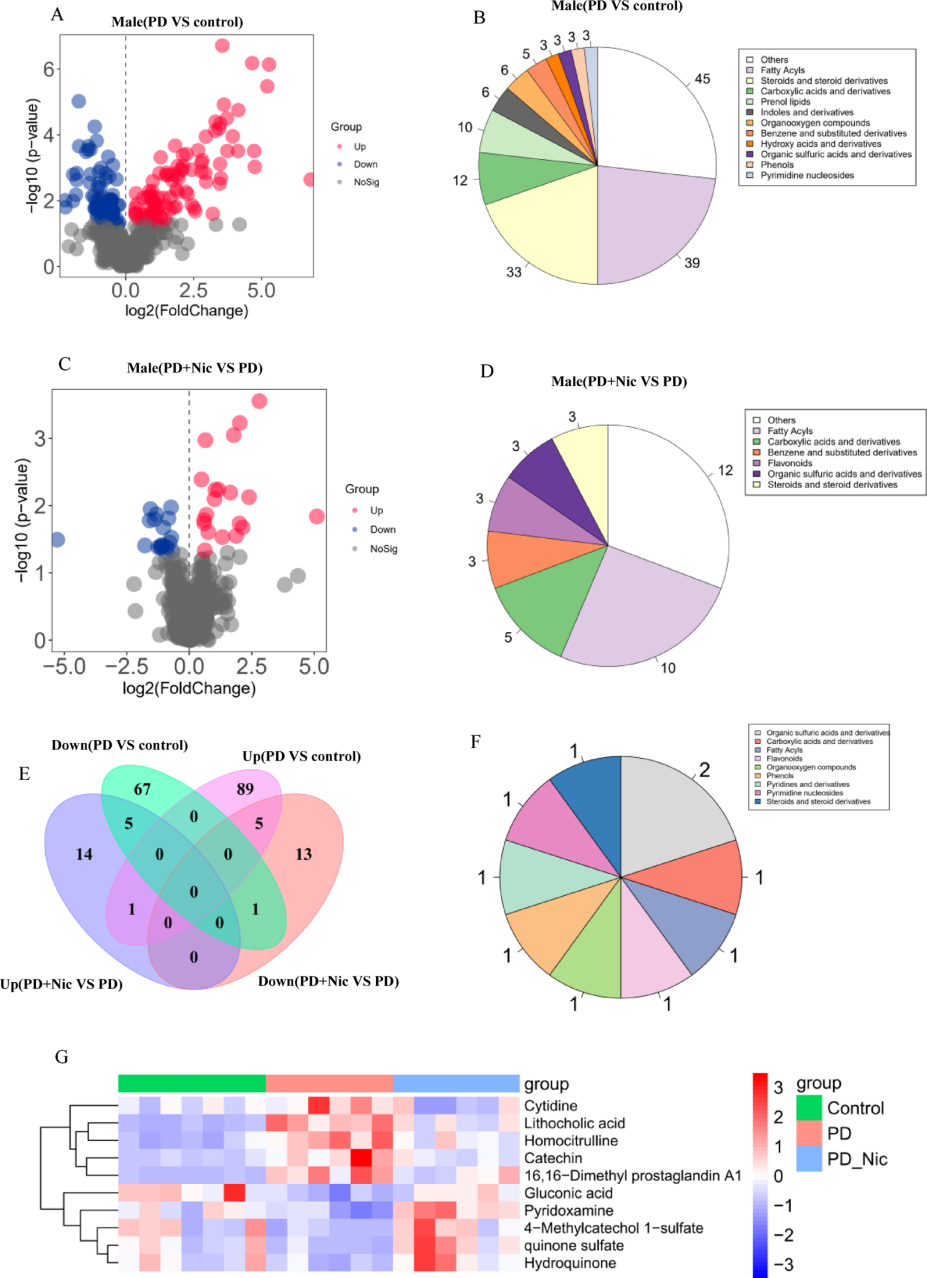
Male-specific metabolic modulation by nicotine in PD rats.** A** Volcano plot of differential metabolites between the PD and control groups. **B** Top perturbed metabolite classes between the PD and control groups. **C** Volcano plot of differential metabolites between the PD+Nic and PD groups. **D** Top perturbed metabolite classes between the PD+Nic and PD groups. **E** Venn diagram of metabolite overlap among groups. **F** Class distribution of reversed metabolites. **G** Heatmap of z-score normalized metabolites demonstrating nicotine-mediated normalization of PD signatures

In males, PD altered 168 metabolites compared with controls (95 upregulated, 73 downregulated; Fig. 6A), with major shifts in fatty acyls, steroids/derivatives, and carboxylic acids/derivatives (Fig. 6B). Nicotine modulated 39 metabolites (20 upregulated, 19 downregulated vs. PD; Fig. 6C), primarily impacting fatty acyls and carboxylic acids/derivatives (Fig. 6D). Only 10 metabolites were reversed by nicotine (5 suppressed upregulated metabolites, 5 elevated downregulated metabolites; Fig. 6E), including organic sulfuric acids/derivatives, carboxylic acids/derivatives, and fatty acyls (Fig. 6F). Specifically, nicotine reduced cytidine, lithocholic acid, catechin, homocitrulline, and 16,16-dimethyl prostaglandin A1 while increasing gluconic acid, 4-methylcatechol-1-sulfate, quinone sulfate, hydroquinone, and pyridoxamine (Fig. 6G).

**Fig. 7:**
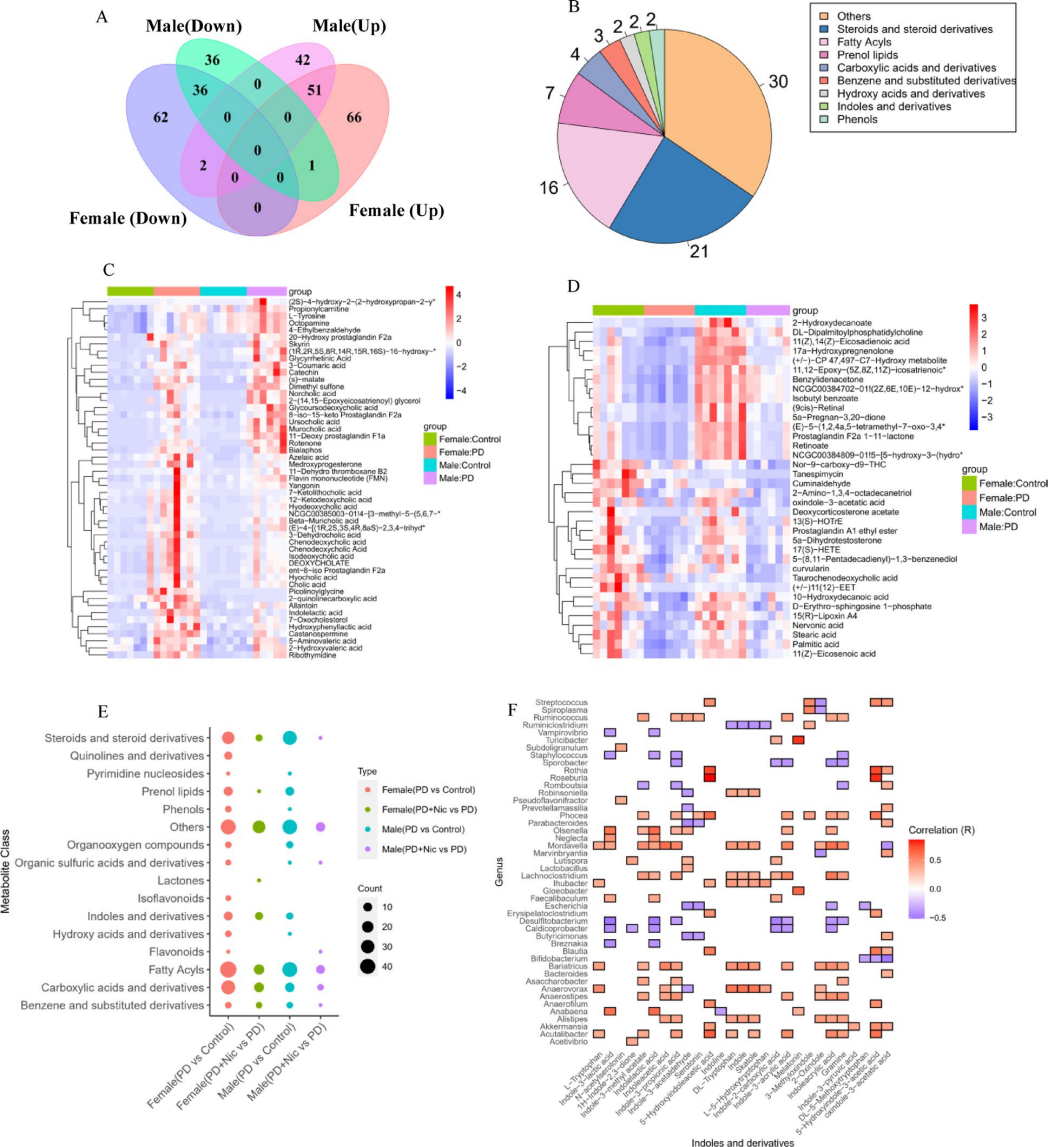
Conserved and sex-divergent metabolic signatures in PD rats. **A** Venn diagram of metabolites dysregulated in both sexes (PD vs. control). **B** Class distribution of common dysregulated metabolites (PD vs. control). **C-D** Sex-stratified heatmaps of conserved metabolic alterations. Metabolite names marked with an asterisk are abbreviated due to length, full names are provided in Supplementary Table S3. **E** Shared metabolic classes modulated by nicotine in both. **F** Pearson correlation heatmap between indole derivatives and gut microbiota genera. All displayed correlations are statistically significant (*p* < 0.05) with color gradients from blue to red indicating negative and positive correlations respectively

Comparative metabolomic analysis identified 87 commonly dysregulated metabolites in PD rats compared with controls across both sexes (51 elevated, 36 reduced; Fig. 7A), predominantly spanning steroids/derivatives, fatty acyls, and prenol lipids (Fig. 7B). Sex-stratified heatmaps visualized these conserved metabolic perturbations (Fig. 7C-D). Among the 21 steroid-related compounds, 15 bile acids and their derivatives were significantly elevated in the PD group, whereas taurochenodeoxycholic acid was uniquely reduced. Crucially, nicotine treatment did not reverse any individual metabolites that were commonly altered in both sexes. However, it significantly modulated several shared metabolic classes in PD rats of both sexes (Fig. 7E), particularly fatty acyls, carboxylic acids/derivatives, and steroids/derivatives. While these classes responded similarly to nicotine in both sexes, indole derivatives exhibited female-predominant modulation. Pearson correlation analysis revealed significant associations between indole derivatives and gut microbiota genera (*p* < 0.05; Fig. 7F). In female PD rats, elevated indole metabolites relative to controls included gramine, indole-2-carboxylic acid, indole-3-acrylic acid, ILA, indole-3-methyl acetate, IPA, and melatonin (Supplementary Table 3), while oxindole-3-acetic acid was significantly decreased. Male PD rats exhibited elevated 1 H-indole-2,3-dione, 2-oxindole, and indolelactic acid, with decreased 5-hydroxyindole-3-acetic acid, N-acetylserotonin, and oxindole-3-acetic acid. Notably, *Mordavella* abundance decreased in males but increased in females (Fig. 4F), showing significant positive correlations with ILA, IPA, and indole-3-acrylic acid (Fig. 7F). Both *Desulfitobacterium* and *Caldicoprobacter* demonstrated reduced abundance exclusively in female PD (Fig. 4F), and exhibited negative correlations with ILA and indole-3-acrylic acid.

## Discussion

Despite increasing recognition of sex differences in PD pathology, sex-specific responses to pharmacological interventions remain largely underexplored. Here, we address this gap using chronic nicotine administration in a rotenone-induced PD rat model and uncover sex-dependent differences in neuroprotective responses across motor, nigral, gastrointestinal, and metabolic domains. Chronic nicotine administration exerted neuroprotective effects in both sexes, with sex-dependent differences in specific functional, gastrointestinal, and metabolic domains. Females demonstrate superior benefits in dynamic motor function recovery and gastrointestinal preservation, accompanied by changes in gut microbiota composition, including the normalization of *Romboutsia*, and reductions in inflammatory markers such as IL-6. Conversely, males exhibited preferential restoration of balance-related motor parameters, potentially reflecting nigrostriatal circuit protection, alongside microbiome-mediated restoration of butyrate producers (*Blautia/Marvinbryantia*), yet retained vulnerability to bile acid dysregulation and attenuated metabolic rescue.

Nicotine-induced motor rescue revealed sex-dependent differences in the severity and pattern of gait impairment in the rotenone-induced PD model, while the overall directional effects of rotenone and nicotine were broadly comparable between males and females. Consistent with the behavioral readouts, rotenone produced robust motor impairment on the rotarod in both sexes, and nicotine significantly improved performance in both males and females (Fig. 1A), supporting a shared protective effect that is not restricted to one sex. Within CatWalk parameters, males exhibited more pronounced postural instability, including a significant increase in base of support and a male-specific increase in step cycle following rotenone exposure (Fig. 1E–F), paralleling the greater postural instability and faster disease progression reported in male patients, which have been linked to lower striatal dopamine binding [34]. Notably, nicotine significantly reduced base of support in males (Fig. 1F), whereas step cycle was not significantly normalized (Fig. 1E), indicating that nicotine’s balance-related benefit was parameter-specific rather than globally restorative across all timing measures [35]. In contrast, females showed clearer nicotine-associated improvements in dynamic gait parameters, with nicotine significantly reducing rotenone-elevated stance time and significantly increasing swing speed relative to PD (Fig. 1B–C). Both sexes also exhibited a significant nicotine-mediated restoration of stride length (Fig. 1D), reinforcing that key aspects of gait hypokinesia are nicotine-responsive across sexes. Together, these findings suggest that nicotine exerts a broadly convergent therapeutic direction on motor dysfunction, while the most statistically robust improvements differ by metric and sex—favoring balance and postural stability in males and temporal and speed-related dynamics in females (Fig. 1B–F). This pattern is consistent with prior reports suggesting that estrogen can enhance nAChR signaling via ERα [34, 36]. It also accords with clinical observations that female PD patients more often exhibit a tremor-dominant phenotype and a slower rate of motor decline, potentially supported by estrogen-associated metabolic advantages and relatively preserved nigrostriatal function [34, 35].

Our findings indicate that nicotine ameliorated gastrointestinal pathology and inflammatory features in both male and female PD rats, with overall improvement observed across sexes (Fig. 4). Notably, the magnitude of improvement was greater in females, as reflected by more pronounced reductions in colon histopathology scores, plasma α-synuclein levels, and IL-6 expression. In contrast, male PD rats exhibited comparable directional changes following nicotine treatment, although these effects were generally smaller and did not reach statistical significance. In addition, male rats exhibited greater susceptibility to α-synuclein accumulation following rotenone exposure in both the gut and the brain (Fig. 2C, 3C). This pattern parallels the male-biased exacerbation of PD endophenotypes observed in LRRK2 G2019S carriers exposed to prodromal intestinal inflammation [25]. Similar sex-associated trends have also been described in experimental models and clinical studies reporting greater α-synuclein burden or related pathological markers in males [37, 38]. Together, these observations support the interpretation that males may be more vulnerable to α-synuclein accumulation along the gut–brain axis [39, 41].

Parallel to histopathological changes, our data reveal sex-specific shifts in gut microbiota composition and their differential restoration by nicotine (Fig. 4F). Both male and female PD rats exhibited a conserved microbial pattern marked by increased *Bifidobacterium* and decreased *Sporobacter*, consistent with clinical observations [42, 43]. Although *Bifidobacterium* is typically considered beneficial, its elevation in PD may reflect medication effects or gut inflammation, especially under COMT inhibitor treatment [42].

Notably, sex-divergent microbial losses were observed: males exhibited reduced abundance of butyrate-producing genera (*Blautia*,* Marvinbryantia*) (Fig. 4G), while females showed decreased *Romboutsia* (Fig. 4H). These shifts carry distinct pathophysiological consequences: *Blautia* depletion compromises butyrate-mediated microglial suppression via RAS-NF-κB inhibition [44], whereas *Romboutsia* loss has been linked to depressive symptoms and neurotransmitter imbalances in PD [45, 46]. Nicotine reversed these alterations in a sex-specific manner, restoring *Blautia* and *Marvinbryantia* in males and *Romboutsia* in females, alongside improvements in colonic α-synuclein pathology and inflammation. These results implicate sex-specific microbial targets as critical mediators of nicotine’s gut–brain axis effects in PD.

Our metabolomic analysis reveals sex-dependent differences in indole derivative profiles in rotenone-induced PD models. In female PD rats, we observed significant elevations in multiple indole metabolites, including ILA, IPA, indole-3-acrylic acid, and gramine, compared with healthy controls (Fig. 5G). These elevations correlated with an increased abundance of *Mordavella* and decreased abundances of *Desulfitobacterium* and *Caldicoprobacter* (Fig. 7F). Critically, ILA and IPA were potent AhR ligands that can activate downstream anti-inflammatory pathways implicated in restoring intestinal barrier integrity and mitigating neuroinflammation [47, 50]. The female-specific elevation of these AhR-activating indoles likely confers neuroprotective benefits, potentially explaining the attenuated PD severity typically observed in females. Conversely, male PD rats exhibited significant reductions in neuroprotective indoles, including 5-hydroxyindole-3-acetic acid and N-acetylserotonin, alongside decreased *Mordavella* abundance (Fig. 4F). This depletion of AhR-activating metabolites may impair anti-inflammatory signaling pathways [51, 53], rendering male PD rats more vulnerable to neuroinflammation and dopaminergic degeneration.

Nicotine treatment exerted differential effects on these metabolic perturbations between sexes. In females, nicotine significantly normalized 51 PD-altered metabolites (Fig. 5E-G), with pronounced suppression of elevated indole derivatives (ILA, IPA, and indoleacrylic acid). Given that ILA and IPA overproduction in female PD rats may reflect compensatory AhR pathway activation against neurodegeneration [47], nicotine’s ability to downregulate these metabolites could represent a restoration of homeostasis rather than the sole suppression of protective signals. In males, nicotine modulated only 10 metabolites (Fig. 6G), failing to reverse the critical deficit in AhR-activating indoles such as 5-hydroxyindole-3-acetic acid and N-acetylserotonin. This limited metabolic efficacy aligns with nicotine’s poorer functional recovery in male PD models and underscores their inherent vulnerability due to impaired indole-mediated AhR signaling.

## Conclusion

These findings underscore the critical interplay between gut microbiota composition, indole metabolism, and AhR activation in determining PD susceptibility and progression in a sex-dependent manner. This mechanistic divergence provides a framework for understanding how sex-associated biological differences may contribute to the higher incidence of PD in males, underscoring the critical need for sex-stratified therapeutic strategies targeting these distinct pathways. Future therapeutic strategies should prioritize sex-specific approaches: boosting indole-producing microbiota or administering AhR ligands in males, while fine-tuning AhR pathway modulation in females. Understanding how sex hormones regulate this microbiota-indole-AhR axis warrants further investigation to develop precision interventions for PD.

## Supplementary Information

Supplementary Material 1.

## Data Availability

Data generated or analyzed during this study are provided in the Supplementary Information files (Supplementary Tables 1–3). All data supporting this study are available from the corresponding author upon reasonable request. 16 S rDNA gene sequence data have been deposited in the SRA database under accession number PRJNA1285081. Metabolomics data have been deposited in the MetaboLights repository under accession number REQ20250702211572.
